# Turn on the super-elastic collision nature of coronal mass ejections through low approaching speed

**DOI:** 10.1038/srep19576

**Published:** 2016-01-21

**Authors:** Fang Shen, Yuming Wang, Chenglong Shen, Xueshang Feng

**Affiliations:** 1 SIGMA Weather Group, State Key Laboratory of Space Weather, Center for Space Science and Applied Research, Chinese Academy of Sciences, Beijing 100190, China; 2 CAS Key Laboratory of Geospace Environment, Department of Geophysics and Planetary Sciences, University of Science and Technology of China, Hefei, Anhui 230026, China; 3 Synergetic Innovation Center of Quantum Information & Quantum Physics, University of Science and Technology of China, Hefei, Anhui 230026, China; 4 Collaborative Innovation Center of Astronautical Science and Technology, Hefei 230026, China

## Abstract

It has been proved from the observations and numerical simulations that the collision between solar coronal mass ejections (CMEs), the largest plasmoids in the heliosphere, could be super-elastic. This finding suggests that the CMEs’ magnetic energy and thermal energy could be converted into kinetic energy through a more efficient way. However CME collisions are not always super-elastic, which means that this distinct property of plasmoids is probably excited conditionally. As the first attempt, we carry out a series of three-dimensional numerical experiments, and establish a diagram showing the dependence of the collision nature on the CME speed and *k*-number, the ratio of the CME’s kinetic energy to the CME’s total energy. It is found that the super-elastic nature of CMEs appears at the relatively low approaching speed, and most of the previous case studies are in agreement with this diagram. Our study firmly advances the understanding of the super-elastic property of plasmoids, and does give us new clues to deeply understand why and how the magnetic energy and/or thermal energy of the colliding plasmoids can be converted into kinetic energy in such an efficient way.

As the largest plasmoid in the heliosphere, coronal mass ejections (CMEs) play extremely important role in space physics and space weather. The dynamic process of CMEs is key information for us to evaluate their geoeffectiveness. When two or more CMEs collide, their shape, velocity, and propagation direction may change significantly^1^,^2^,^3^,^4^,^5^,^6^,^7^,^8^,^9^,^10^,^11^,^12^, and the colliding CMEs will probably form the well-known multiple-magnetic-cloud structure, which may result in enhanced geoeffectiveness as compared to single CME events^13^,^14^.

In 2012, C. Shen *et al.*^12^ presented an in-depth analysis of a collision between two CMEs in the heliosphere observed by the Solar TErrestrial RElations Observatories (STEREO)^15^ during 2–8 November 2008. It for the first time revealed that the collision of such large plasmoids could be super-elastic, which previously was only found in granular physics^16^,^17^,^18^. The super-elastic process means that the total kinetic energy of the colliding system increases after the collision. Soon, F. Shen *et al.*^19^ confirmed the result through three-dimensional (3D) MHD simulations of the event, and further suggested that the extra kinetic energy gain mainly comes from the magnetic energy of the CMEs. However, those simulations only focused on the CMEs similar to those in the 2008 November event. It is not clear under which conditions the collision between CMEs is super-elastic.

On the other hand, more observational and simulation cases of the CME collisions were studied recently^6^,^10^,^20^,^21^,^22^. In their studies, the authors used 1D head-on collision theory to briefly analyze and discuss the collision nature. Unfortunately, none of those results supported the super-elastic nature of the CME collision. Why is there such a large disparity between the work by C. Shen *et al.* and those of others? We find that the approaching speed of CMEs in most of those cases are much larger than that in C. Shen *et al.* study. It brings us the motivation to invetigate how the CME’s kinematic parameters, e.g., the approaching speed, influence the collision nature of plasmoids. Here, we carry out numerical experiments to parametrically study the collision of CMEs with different speeds.

## Five test cases

In this work, we focus on the head-on collision of two identical CMEs, which are modeled as magnetized plasmoids^11^,^23^ (refer to the section of ‘Methods’ for details). We first test five cases with different initial speeds of the two CMEs, *V*_*CME*1_ and *V*_*CME*2_, which are the average speeds (see ‘Methods’ for explanation) of the CME plasmas. We define the difference between them, Δ*V*_*c*_ = *V*_*CME*2_ − *V*_*CME*1_, as the approaching speed of the two CMEs. Please note that the approaching speed right before the collision is different from Δ*V*_*c*_ defined here, as the CME speed will be modified due to the interaction with the ambient solar wind. In these five cases, we fix *V*_*CME*1_ to 200 km s^−1^ but choose 200, 400 and 800 km s^−1^ as Δ*V*_*c*_ for Case 1 to 3, respectively, and fix Δ*V*_*c*_ to 200 km s^−1^ but set *V*_*CME*1_ to be 600 and 800 km s^−1^ for Case 4 and 5, respectively.

We let the two CMEs collide around 20 ± 5 solar radii (*R*_*S*_) by adjusting the separation time of the launches of them, and minimize the magnetic reconnection between them by reversing the polarity of one CME. Table 1 lists the common and different initial parameters of the CMEs for these test cases. In order to ensure that the two CMEs fully collide before they reach the outer boundary of the computational domain, we use the different range in radial direction for the different cases. The detailed description of our numerical scheme and simulation model has been given in the section of ‘Methods’ at the end of this paper.

For each case, we run a non-collision case as a reference case, in which the second CME is ejected into the opposite direction to make sure that there is no collision. By comparing the collision and non-collision results, we can learn how the energies change and whether the collision is super-elastic, elastic or inelastic.

**Figure 1:**
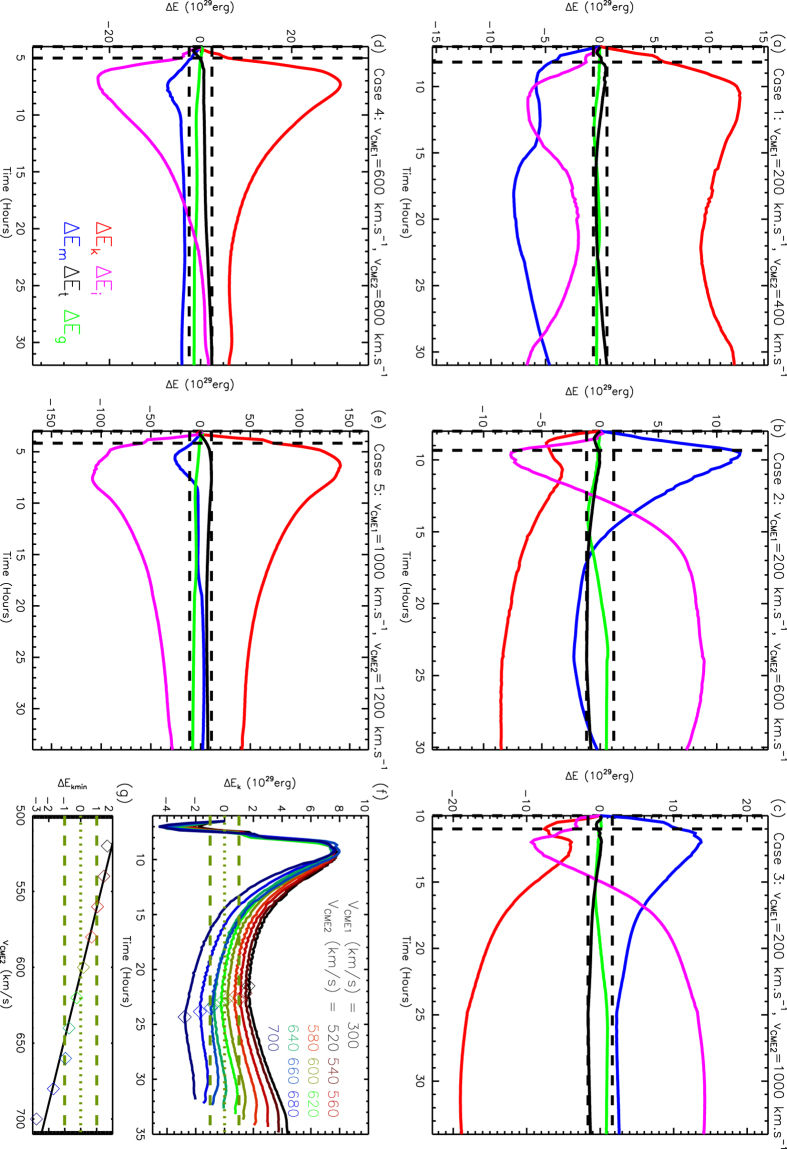
Panel (**a–e**) show the energy differences between the collision and non-collision results for the five test cases. The vertical dashed lines in the panels mark the start time of the collisions. The horizontal lines give the numerical error. Panel (**f**) displays how the difference of the kinetic energy changes with *V*_*CME*2_ for the case of *V*_*CME*1_ = 300 km s^−1^. The diamonds indicate Δ*E*_*kmin*_ which we used to determine the collision nature. Panel (**g**) shows the dependence of Δ*E*_*kmin*_ on *V*_*CME*2_.

Figure 1a–e show the energy differences between the collision and non-collision simulation results for all the five cases. The dashed horizontal lines indicate the level of numerical error, which is determined by the largest difference of total energy, Δ*E*_*t*_, during the simulation interval. As we found before^19^, the gravitational energy is trivial in the CME collision process, because Δ*E*_*g*_ in all the five case is smaller than the numerical error. For all the other energies, the differences are significantly larger than the error and thought to be physically meaningful.

We do not know when the collision ends exactly, because the CMEs have become too diffusive to be distinguished when they are far away from the Sun. But from Fig. 1, we may find that the kinetic energy difference, Δ*E*_*k*_, usually reaches a minimum value after the contact of two CMEs and then rises back gradually. The later increase is probably due to the solar wind that may accelerate the slow CME or the second CME which becomes slow after the collision. This phenomenon is clearer for slow cases as revealed in Fig. 1a,f. In the suplementary information of C. Shen *et al.* paper^12^, it has been shown that the efficiency of the solar wind to change the CME speed is much smaller than (about 6.5% of) that of the CME collision. Thus, we think that the collision process is almost finished when the solar wind influence on the CME kinematics gets relatively significant. Based on this idea, we believe that the collision process is fully covered by the computational domain in our cases, and use the minimum value of Δ*E*_*k*_ after the two CMEs beginning to contact to determine the nature of collision as illustrated by the diamonds in Fig. 1f. Δ*E*_*kmin*_ > 0 means a super-elastic collision, and Δ*E*_*kmin*_ < 0 an inelastic collision or other processes, e.g., a merging process.

From Fig. 1a, we find that the difference of the kinetic energy, Δ*E*_*k*_, increases to about 1.3 × 10^30^ erg in 3.5 hours since the launch of the second CME, then decreases back to about 10^30^ erg and slowly returns to about 1.2 × 10^30^ erg. The value of Δ*E*_*kmin*_ is about 10^30^ erg, which clearly reveals that the collision of the two CMEs is super-elastic. Meanwhile, the both magnetic and thermal energies decreases, suggesting that the extra kinetic energy gain is converted from them. These results are quite similar to those we obtained in the work of ref. 19, as the CME parameters in this case are very close to that.

Figure 1b,c show the energies difference for Case 2 and Case 3. It is found that Δ*E*_*k*_ drops below zero significantly in both cases, suggesting an inelastic process. The lost kinetic energy during the collision mostly goes into the thermal energy. The collision causes temporary gain of additional magnetic energy. In Case 2, the magnetic energy even loses a little bit. Figure 1d,e are similar. After the launch of the second CME, Δ*E*_*k*_ increases first and then decreases, but always stays above the line of zero-change, suggesting a super-elastic collision. Different from Case 1, the kinetic energy gain in both cases are mostly from the thermal energy.

One may notice that the energy difference appears before the collision for some cases. We think that it is because the conditions of the background solar wind before the second CME in the collision and non-collision cases are not identical. In Case 1, 4 and 5, Δ*E*_*k*_ increases at the beginning of the launch of the second CME. It is probably because the second CME needs to consume more kinetic energy to sweep up the slower solar wind ahead of it in the non-collision case than that in the collision case, in which the ambient slower solar wind may be partially removed by the first CME^4^. However, in Case 2 and 3, Δ*E*_*k*_ decreases at the beginning. In these two cases, the first CME is slower than the background solar wind and the second CME is much faster than the first one and the background solar wind. Under this condition, the background solar wind ahead of the second CME in the collision cases becomes much more disturbed, in which the second CME might consume more kinetic energy, than that in the non-collision cases.

**Figure 2:**
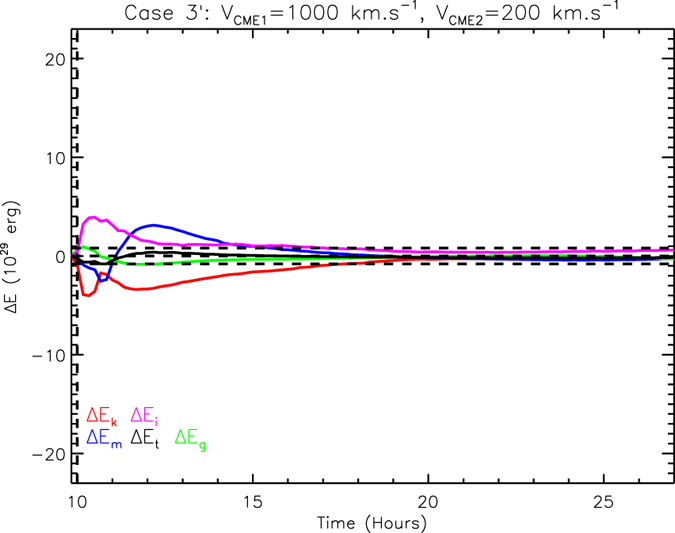
A control case for Case 3, suggesting that the energy differences caused by the different background solar wind ahead of the second CME are temporary.

Such a difference in the kinetic energy before the collision is temporary, and may not be considered in the identification of the collision nature. This can be seen from a control experiment, Case 3’, in which we exchange the launch order of the two CMEs in Case 3 to make sure that the two CMEs cannot collide even if they propagate along the same direction. The differences of the energies for Case 3’ are plotted in Fig. 2. It is found that all the energy differences vanish before 20 hours. The time of Δ*E*_*k*_ reaching the minimum value is after 20 hours for all the cases in our study.

## Search the boundary between super-elastic and inelastic

The above analysis has shown that the collision nature clearly depends on the CME speed, and some collisions are super-elastic whereas some are inelastic. The boundary between them is elastic collision. Hereafter, we try to seek the critical approaching speed, Δ*V*_*c*_, to produce the elastic collision for different initial speed of CME1, *V*_*CME*1_, of 200, 300, 400, 500, 600 and 650 km s^−1^. For any given *V*_*CME*1_, we scan *V*_*CME*2_ with an increasing step of 20 km s^−1^ and calculate the differences of energies until Δ*E*_*kmin*_ almost reaches zero, i.e., the elastic collision is obtained.

For example, for the case of *V*_*CME*1_ being 300 km s^−1^, we make ten numerical tests by changing *V*_*CME*2_ from 520 km s^−1^ to 700 km s^−1^. The color-coded curves in Fig. 1f show the curves of Δ*E*_*k*_ at *V*_*CME*1_ = 300 km s^−1^ and *V*_*CME*2_ = 520, 540, 560, 580, 600, 620, 640, 660, 680 and 700 km s^−1^. It is clearly shown in Fig. 1g that Δ*E*_*kmin*_ monotonously decreases as the approaching speed increases. The collision with the smaller approaching speed tends to be super-elastic. When *V*_*CME*2_ = 600 km s^−1^, i.e., the approaching speed Δ*V*_*c*_ = 300 km s^−1^, the value of Δ*E*_*kmin*_ is almost zero, and we think that this case presents a nearly elastic collision. The uncertainty in Δ*V*_*c*_ is 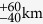 s^−1^, which is defined according to the numerical error as denoted by the horizontal dashed lines in Fig. 1f,g.

**Figure 3:**
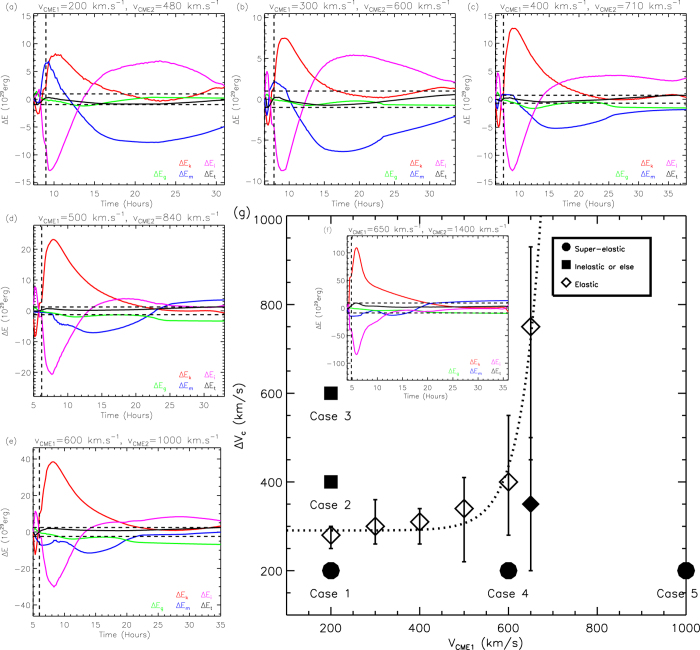
Panel (**a–f**) show the energy differences for six critical cases, in which the value of Δ*E*_*kmin*_ is almost zero. Panel (**g**) exhibits the five test cases (squares for inelastic and dots for super-elastic) and the critical cases (diamonds) in the (*V*_*CME*1_, Δ*V*_*c*_) space. The dotted line shows the exponential fitting to the open diamonds.

By using this method, we find all the critical approaching speeds for the selected values of *V*_*CME*1_, which are 280, 300, 310, 340, 400 and 750 km s^−1^, respectively. The differences of energies for these critical cases are shown in Fig. 3a–f. Looking at the energy exchanges after the simulation time *t* = 15 hours, there is extra magnetic energy notably going into thermal energy compared with the non-collision results in the first three critical cases, while in the other three critical cases, the extra energy conversion occurs mainly between the magnetic energy (critical case d and f) or thermal energy (critical case e) and the gravitational energy. No matter which case is, the extra energy exchange at the end of simulation time is about or even less than 5 × 10^29^ erg, occupying a very small fraction of the CME’s total energy. Figure 3g shows the dependence of the collision nature on the speed. Previous Case 1 to 5 are marked on it by black dots for super-elastic collisions and black squares for inelastic collisions. The six critical cases are plotted as the open diamonds with the error bars. Apparently these critical points follow an exponential function, 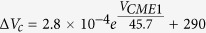, as denoted by the dotted line, below which the collision is super-elastic.

Energy conversion is the fundamental process in a collision, and how much non-kinetic free energy could be converted into kinetic energy, we think, is a key factor behind the speed that may influence the collision nature. Thus, we investigate the ratio of the CME’s kinetic energy to its total energy, which we define as *k*-number. It measures the balance between the kinetic energy and non-kinetic energies of a CME. A *k*-number of 0.5 means that the CME’s energy is equally partitioned into the kinetic energy and other energies. In our previous numerical experiments, the *k*-number varies along with the speed of the CME. According to Table 1, we estimate that the value of the *k*-number increases from less than 0.20 to more than 0.85 as *V*_*CME*_ increases from 200 to 1200 km s^−1^. This reflects a change from a non-kinetic-energy dominated CME to a kinetic-energy dominated CME. One can see from Fig. 3g that the critical approaching speed increases gradually with the increasing *V*_*CME*1_ if the first CME is non-kinetic-energy dominated, and increases quickly when the CME becomes kinetic-energy dominated.

We now investigate what happens if the CME’s *k*-number is lowered by adding another case of *V*_*CME*1_ = 650 km s^−1^. By increasing the magnetic field strength and temperature, we let *k*-number of the first CME be about 0.46. This number is almost the same as that in the critical case of *V*_*CME*1_ = 400 km s^−1^, but much smaller than the *k*-number, 0.67, in the original case of *V*_*CME*1_ = 650 km s^−1^. As before, the parameters of the second CME are exactly same as the first one except the speed. The numerical experiments suggest that the critical approaching speed is about 350 km s^−1^ with the uncertainty of about ±150 km s^−1^ as marked by the filled diamond in Fig. 3g. Clearly, it is significantly different from the original critical case of *V*_*CME*1_ = 650 km s^−1^, and also different from the critical case of *V*_*CME*1_ = 400 km s^−1^ though the values of the *k*-number of their first CMEs are almost the same. This test suggests that the critical approaching speed, or the boundary between super-elastic and inelastic, is influenced by both CME speed and *k*-number.

## Diagram of the collision nature

**Figure 4:**
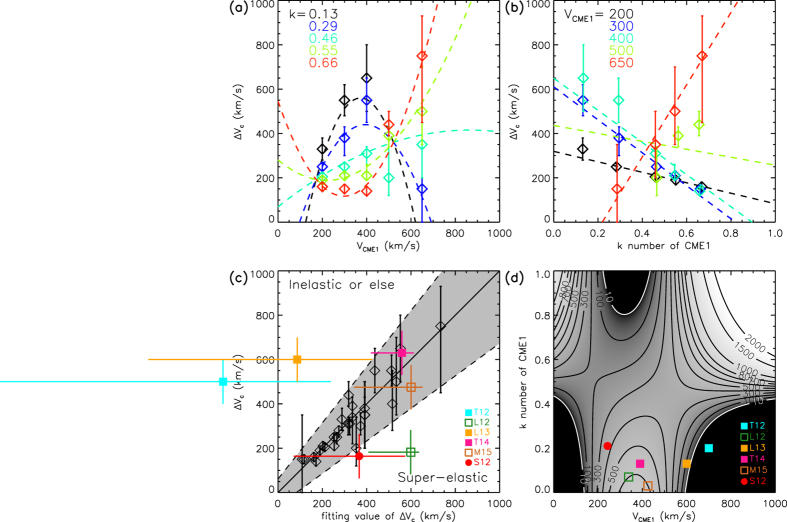
Panel (**a**) shows how Δ*V*_*c*_ varies with *V*_*CME*1_. The different colors indicate different values of the *k*-number of the first CME. The dashed lines are the quadratic fittings. Panel (**b**) shows how Δ*V*_*c*_ varies with the *k*-number of the first CME. The different colors indicate different value of *V*_*CME*1_. The dashed lines are the linear fittings. Panel (**c**) presents the correlation between the critical approaching speeds and their fitting values. The shadow region denotes the 3-sigma uncertainty. It is super-elastic below the shadow region, and inelastic above the shadow region. Panel (**d**) shows the surface of the critical approaching speed in the (*V*_*CME*1_, *k*) space. Previously studied events are marked by color-coded symbols in Panel (**c**,**d**). The dot for super-elastic and the squares for inelastic. The filled symbol means that the event is in agreement with the diagram (Panel **d**) derived from our numerical experiments, and the open symbol means a more or less disagreement.

To give a complete picture of how the collision nature influenced by the CME’s kinetic parameters. We further search the following critical cases: the speed of the first CME is 200, 300, 400, 500 and 650 km s^−1^, respectively, and the *k*-number of the first CME is about 0.13, 0.29, 0.46, 0.55 and 0.66, respectively. The critical approaching speed can be obtained except for the cases of (*V*_*CME*1_, *k*) = (500, 0.29), (500, 0.13) and (650, 0.13). Figure 4a,b show the critical approaching speed as a function of the first CME’s speed and *k*-number, respectively, based on these critical cases. For a given *V*_*CME*1_ ≤ 400 km s^−1^, a larger *k*-number requires a smaller critical approaching speed, whereas for a given *V*_*CME*1_ ≥ 500 km s^−1^, the trend is reversed. Further, it could be found that Δ*V*_*c*_ is a linear function of *k* for any given *V*_*CME*1_, and roughly follows a parabolic function of *V*_*CME*1_ for any given *k*. The parabolas open up when the *k*-number is larger than 0.5, and seemingly open down when the *k*-number is smaller than 0.5.

Based on these facts, we find that these critical cases as well as the critical cases presented in the previous section can be well fitted by the function


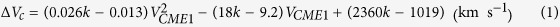


or


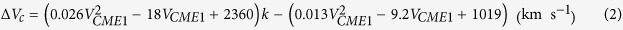


as shown in Fig. 4c. The correlation coefficient between the two sets of Δ*V*_*c*_ is as high as 0.94, and the shadow region gives the 3-sigma uncertainty of the fitting. The collision below the shadow region is super-elastic and that above the region is inelastic. The fitting curves are also superimposed onto Fig. 4a,b as the dashed lines for comparison. It can be found that most data points are generally in agreement with the corresponding fitting curves except the green ones in Fig. 4b, implying that 500 km s^−1^ or so is a very special value for *V*_*CME*1_. From Eq. 2, we can find that except *V*_*CME*1_ = 500 km s^−1^, *V*_*CME*1_ = 170 km s^−1^ is another special value. Both of them may vanish the first term on the right-hand side of Eq. 2. Possible reasons why the two speeds are so special will be discussed later in the section of ‘Summary and discussion’. On the other hand, *k* = 0.5 is also a special value as it makes Eq.1 reduce from the parabolic function to a linear function. It is clear that this special *k*-number distinguishes the kinetic-energy dominated and the non-kinetic-energy dominated CMEs.

Figure 4d presents the fitting surface of the critical approaching speed in the (*V*_*CME*1_, *k*) space. It suggests that an approaching speed below the surface should cause a super-elastic collision. From this diagram, one may expect that it is hard to achieve a super-elastic collision in the three dark regions, in which the critical approaching speed is less than 10 km s^−1^. The upper-right corner seems to be the most favorable region to have a super-elastic collision though the uncertainty in that region is much more significant than that in other regions.

## Comparison with previous observational and simulation cases

**Figure 5:**
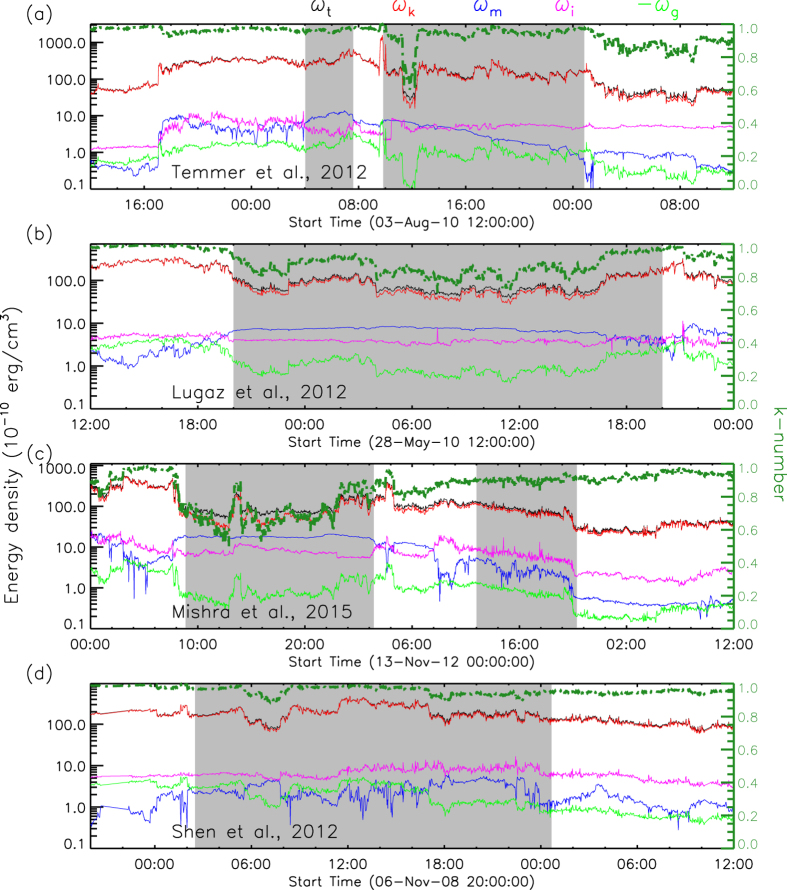
Energy densities along the observational path derived from the *in situ* measurements of the Wind spacecraft at 1 AU. The *k*-number is plotted in green lines and scaled by the right vertical axis. The CMEs are marked by the shadow regions.

Now we have established the diagram from the numerical experiments to determine if a collision is super-elastic or inelastic based on the CME’s initial speed and *k*-number. For our previously studied observational case^12^ (labelled as S12), we may derive the energy densities and the *k*-number of the first CME along the observational path according to the *in situ* measurements at 1 AU, as shown in Fig. 5d. The average *k*-number of the CME, marked by the shadow region, is therefore estimated to be about 0.96 at 1 AU. Since the established diagram is for the parameters at about 2 *R*_*S*_, where the CME initially introduced, we further convert the *k*-number at 1 AU to that at 2 *R*_*S*_, which is about 0.21. The detailed description of how to derive the *k*-number could be found in the section of ‘Methods’. Recall the initial speed of the CME is about 243 km s^−1^ and the approaching speed is about 164 km s^−1^, this case is in agreement with the diagram as indicated by the red dot in Fig. 4c,d.

We further test the diagram with five observational and simulation cases presented by others in the previous literatures. For convenience, we call them Case T12 (ref. 10), L12 (ref. 6), L13 (ref. 21), T14 (ref. 20) and M15 (ref. 22). Except that Case L13 is a simulation case, all the other cases are observational, and particularly three of them (T12, L12 and M15) have *in situ* observations, which suggest that the average *k*-numbers of the first CMEs are about 0.96, 0.86, 0.70, respectively, at 1 AU (see Fig. 5a–c), or about 0.20, 0.07, 0.03 after being converted to the values at 2 *R*_*S*_. Since there is no *in situ* observations of Case T14, we tentatively assume that it has a *k*-number of 0.13, the mean value of the above cases. Case L13 actually consists of four numerical simulations of different orientations between two colliding CMEs. Since the speeds of the two CMEs in those simulations are the same, they correspond to only one point in our diagram. Also we assume that the *k*-number of the first CME in Case L13 is the mean value, 0.13. All these five cases are marked by color-coded squares in Fig. 4c,d as they were claimed to be inelastic collisions. It should be noted that the CMEs’ initial speeds, i.e., the CME speeds at several solar radii rather than the speeds right before the collision, of these cases should be used in our diagram. For Case M15, the authors did not give the CMEs’ speeds near the Sun, and therefore we roughly estimate them according to Fig. 6 of their paper. The error bars are calculated by assuming an uncertainty of ±100 km s^−1^ in the velocity and an uncertainty of ±50% in the *k*-number.

It could be found from Fig. 4c that, in all the five independent cases, Case T12 and L13 are in agreement with the diagram, Case T14 and Case M15 locate in the shadow region where the determination of the collision nature is somewhat ambiguous, and the other case L12 is contrary. Plus our observational case S12, which is consistent with the diagram too, we find that there is only one definitely exceptional case in the six cases. Besides, we should note that the comparison between our diagram and these independent cases is preliminary because (1) the CMEs’ kinetic parameters in these cases suffer from large uncertainties and (2) some collision conditions, e.g., the approaching angle, and collision distance, etc., may differ from those used in our diagram. A more precise revisit to those events is worth to be done in a follow-up work.

## Summary and Discussion

We have quantitatively studied the influence of the speed and *k*-number on the CMEs’ collision nature through numerical experiments. The derived diagram, Fig. 4d, is particularly useful in roughly estimating the collision nature as long as we know the values of the CMEs’ speeds and the *k*-number, which are all possibly obtainable from observations. Concretely speaking, a smaller approaching speed is more favorable for a super-elastic collision, and there is a surface of the critical approaching speed in the (*V*_*CME*1_, *k*) space to separate the super-elastic and inelastic (including the merging) processes. The critical approaching speed is linearly correlated with the *k*-number and roughly quadratically correlated with the first CME’s speed.

We mentioned before that 500 and 170 km s^−1^ are two special values of *V*_*CME*1_. From Fig. 4b, we can see that the linear correlation between Δ*V*_*c*_ and *k*-number is positive/negative for the cases of *V*_*CME*1_ above/below 500 km s^−1^. A similar inference could be obtained for *V*_*CME*1_ ≈ 170 km s^−1^ according to Eq. 2. This is probably related to the presence or absence of the CME-driven shock. In our simulations, the background solar wind has a speed of 316–461 km s^−1^. Considering a fast-mode magnetosonic wave speed of about hundreds kilometers per second near the Sun or tens kilometers per second near 1 AU, the condition of a CME to right drive a shock ahead is travelling with a speed of about 500 km s^−1^. Similarly, a reverse shock will appear behind a CME if the CME propagated much slowly, say less than 170 km s^−1^. If this is ture, the CME-driven shock, no matter where it locates, not only significantly changes the dynamics of a CME, but also has influence on the nature of the collision between CMEs. The role of shocks played during the CME-CME interaction was discussed in previous studies^5^. However, how the shock changes the interaction process is still far from clear. In this study, we just treat a shock as a part of its associated CME, and it will not change the diagram shown in Fig. 4. A deeper analysis about the role of shocks might be able to be done through the simulations, but needs more work.

Besides, in this study we ignore many other influence factors, such as the magnetic field reconnection, the approaching angle, the collision distance and the background solar wind. As the first kind of this study, however, it does largely advance our understanding of the dynamic behavior of plasmoids, and shed the light on the underlying physical mechanisms of the collisions of plasmoids and the energy exchange/conversion. The most important implication here is that that the super-elastic collision nature is probably an intrinsic property of any expanding magnetized plasmoids, e.g., CMEs, and could be turned on through a low approaching speed. So far we do not explicitly know what the physical mechanism governs the super-elastic process. A rough picture, we believe, is that a low approaching speed allows the two colliding CMEs to fully interact, and then the CME plasmas can do work efficiently through expansion, during which the magnetic energy and thermal energy are converted into kinetic energy.

## Methods

### Three-dimensional MHD numerical model

The numerical scheme used in this study is a 3D corona interplanetary total variation diminishing (COIN-TVD) scheme in a Sun-centered spherical coordinate system (*r*, *θ*, *φ*) with the *r*-axis in the ecliptic plane^24^,^25^,^26^,^27^. The computational domain in *θ* and *φ* directions covers −89° ≤ *θ* ≤ 89° and 0° ≤ *φ* ≤ 360°. We use different ranges in the radial direction for the different cases to make sure that the collision is fully developed in the computational domain: 1*R*_*S*_ ≤ *r* ≤ 100*R*_*S*_ in Case 1 and 2, 1*R*_*S*_ ≤ *r* ≤ 150*R*_*S*_ in Case 3 and 4, and 1*R*_*S*_ ≤ *r* ≤ 220*R*_*S*_ in Case 5. The grid size is uniform in the azimuthal direction with Δ*φ* = 2°. In order to obtain a better computational resolution, we use non-uniform grids in the radial and meridional directions. The radial grids are set as *r*_0_ = 1*R*_*S*_, Δ*r*_0_ = *s* × *r*_0_, *r*_*i*_ = *r*_*i*−1_ + Δ*r*_*i*−1_, Δ*r*_*i*_ = *s* × *r*_*i*_, where *s* = *π*/200. The meridional grids are set as 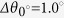, 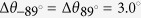, with a constant increase in Δ*θ* from *θ* = 0° to *θ* = ±89°.

To construct a steady background solar wind, we use the potential field extrapolated from the line-of-sight of photospheric magnetic field of Carrington rotation 2076 from the Wilcox Solar Observatory (WSO) and the Parker’s solar wind solution as the initial magnetic field and velocity, apply the momentum conservation law and adiabatic process to obtain the initial density and temperature, and then run our MHD code to reach a steady state. The projected characteristic boundary conditions^28^,^29^,^30^ are adopted at the lower boundary. We use the same background solar wind for all the cases simulated in this study.

The CMEs are modeled as high-density, -temperature and -velocity magnetized plasm blobs^11^,^23^. In the plasma blob model, a CME can be launched with a given propagation velocity, direction, density, temperature, magnetic field strength and magnetic polarity. The detailed formulae are given below


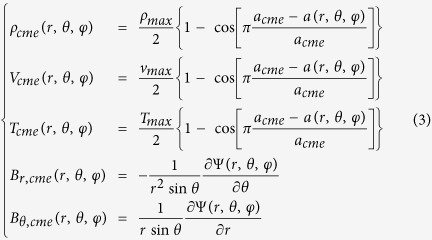


where *a*_*cme*_ is the radius of the initial plasma blob, *a*(*r*,*θ*,*φ*) denotes the distance from the center of the initial plasma blob, *ρ*_*max*_, *v*_*max*_ and *T*_*max*_ are the maximum density, radial velocity and temperature in the plasma blob, respectively, and the magnetic flux function Ψ is given by


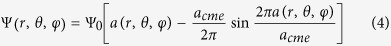


The parameter, *V*_*CME*_, used to characterize the CME speed in the main text is the average value of the velocity of the CME plasma, which is approximate 1/3 of *v*_*max*_. The CME is introduced into the computational domain by directly inserting it into the background solar wind medium with its center at *r* = 2*R*_*S*_ as the following


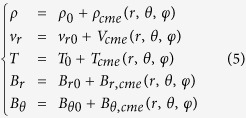


### Infer the *k*-number from *in-situ* solar wind measurements

We use the measurements of the interplanetary magnetic field and solar wind plamsa from Wind spacecraft^31^,^32^ to infer the energy density, i.e., the energy per unit volume, of the events of interest at 1 AU. Wind spacecraft provides the data of the magnetic field strength, **B**, the speeds of protons and electrons, *v*_*p*_ and *v*_*e*_, the number densities of protons and electrons, *n*_*p*_ and *n*_*e*_, and the temperature of protons and electrons, *T*_*p*_ and *T*_*e*_. Based on these parameters, we derive the energy densities by using the following formulae


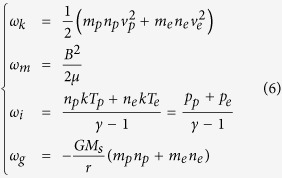


in which *m*_*p*_ = 1.67 × 10^−27^ kg and *m*_*e*_ = 9.1 × 10^−31^ kg are proton and electron masses, respectively, *μ* = 4*π* × 10^−7^ H m^−1^ is the permeability of free space, *k* = 1.38 × 10^−23^ J K^−1^ is the Boltzmann constant, *γ* = 5/3 is the adiabatic index for monoatomic ideal gases, *G* = 6.67 × 10^−11^ m^3^ s^−2^ kg^−1^ is the gravitational constant, *M*_*S*_ = 1.99 × 10^30^ kg is the mass of the Sun, and *r* is the distance from the solar center. The total energy density, *ω*_*t*_, is just the sum of the above energies and the *k*-number is 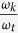. In the study, we use the average value of the *k*-number during the passage of the CME of interest. Here the contribution of minor ions is not considered.

To further infer the value of *k*-number at 2 *R*_*S*_ from the value of the *k*-number at 1 AU, we assume that (1) the density is proportional to *r*^−3^, (2) the temperature is proportional to *r*^−1^ as the erupted plasma near the Sun is typically more than 10^6^ K (ref. 33) and cools down to the order of 10^5^ K at 1 AU, and (3) the speed does not change too much compared to the above parameters. Then, we have


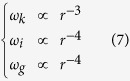


The magnetic energy of a CME decreases by following *r*^−1^ when it propagates away from the Sun^34^,^35^, thus the magnetic energy density follows


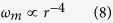


With these scaling laws, we may infer the value of the *k*-number at any given distance as long as its value at 1 AU is known. It should be noted that the *k*-number is obtained based on the measurements along the observational path, and may not precisely reflect the real *k*-number of the whole CME, and the scaling laws applied here are very simplified. Thus, the inferred *k*-number is just an approximation of the real *k*-number, and an uncertainty of ±50% is taken into account (see Fig. 4c).

## Additional Information

**How to cite this article**: Shen, F. *et al.* Turn on the super-elastic collision nature of coronal mass ejections through low approaching speed. *Sci. Rep.* **6**, 19576; doi: 10.1038/srep19576 (2016).

## Figures and Tables

**Table 1:** Initial parameters of the CMEs and background solar wind.

	Direction	*R*	*B*	*n*	*T*	*Em*	*Ei*	*Eg*	*Vsw*	
Common par.	N11W18	*R*_*S*_	×10^5^ nT	×10^7^ cm^−3^	×10^5^ K	×10^31^ erg			km s^−1^	
		0.5	1.47	4.0	5.0	1.50	1.37	−0.64	316 ~ 461	
Other par.	Case 1		Case 2		Case 3		Case 4		Case 5	
	CME1	CME2	CME1	CME2	CME1	CME2	CME1	CME2	CME1	CME2
*V*_*CME*_ (km s^−1^)	200	400	200	600	200	1000	600	800	1000	1200
*E*_*k*_ (×10^31^ erg)	0.513	1.83	0.513	3.44	0.513	9.13	3.44	5.96	9.13	12.9
*E*_*t*_ (×10^31^ erg)	2.74	4.06	2.74	5.67	2.74	11.36	5.67	8.19	11.36	15.13
*t*_*s*_ (hours)	7		8		10		4		3	

The top three rows list the common initial parameters and the other rows list the different initial parameters. For the common initial parameters, from the second column to the right, they are the propagation direction, radius, magnetic field, number density, temperature, the magnetic, thermal and gravitational energies, and the background solar wind speed along the direction at 18 and 220 *R*_*S*_, respectively. For different initial parameters, from the top to bottom, they are the CME’s initial speed, kinetic energy, total energy and the separation time between the launches of the two successive CMEs.

## References

[b1] Reiner M. J. *et al.* Constraints on coronal mass ejection dynamics from simultaneous radio and white-light observations. Astrophys. J. 590, 533–546 (2003).

[b2] Farrugia C. & Berdichevsky D. Evolutionary signatures in complex ejecta and their driven shocks. Ann. Geophys. 22, 3679–3698 (2004).

[b3] Wang Y., Zheng H., Wang S. & Ye P. MHD simulation of the formation and propagation of multiple magnetic clouds in the heliosphere. Astron. & Astrophys. 434, 309–316 (2005).

[b4] Lugaz N., Manchester, I., W. B. & Gombosi T. I. Numerical simulation of the interaction of two coronal mass ejections from Sun to Earth. Astrophys. J. 634, 651–662 (2005).

[b5] Lugaz N., Vourlidas A. & Roussev I. I. Deriving the radial distances of wide coronal mass ejections from elongation measurements in the heliosphere application to CME-CME interaction. Ann. Geophys. 27, 3479–3488 (2009).

[b6] Lugaz N. *et al.* The deflection of the two interacting coronal mass ejections of 2010 may 23-24 as revealed by combined *in site* measurements and heliospheric imaging. Astrophys. J. 759, 68(13pp) (2012).

[b7] Hayashi K., Zhao X.-P. & Liu Y. MHD simulation of two successive interplanetary disturbances driven by cone-model parameters in IPS-based solar wind. Geophys. Res. Lett. 33, L20103 (2006).

[b8] Xiong M., Zheng H., Wu S. T., Wang Y. & Wang S. Magnetohydrodynamic simulation of the interaction between two interplanetary magnetic clouds and its consequent geoeffectiveness. J. Geophys. Res. 112, A11103 (2007).

[b9] Wu C.-C. *et al.* Three-dimensional global simulation of multiple ICMEs interaction and propagation from the Sun to the heliosphere following the 2528 October 2003 solar events. Adv. in Space Res. 40, 1827–1834 (2007).

[b10] Temmer M. *et al.* Characteristics of kinematics of a coronal mass ejection during the 2010 August 1 CME-CME interaction event. Astrophys. J. 749, 57 (2012).

[b11] Shen F. *et al.* Three-dimensional MHD simulation of two coronal mass ejections’ propagation and interaction using a successive magnetized plasma blobs model. J. Geophys. Res. 116, A09103 (2011).

[b12] Shen C. *et al.* Super-elastic collision of large-scale magnetized plasmoids in the heliosphere. Nature Phys. 8, 923–928 (2012).

[b13] Wang Y. M., Wang S. & Ye P. Z. Multiple magnetic clouds in interplanetary space. Sol. Phys. 211, 333–344 (2002).

[b14] Wang Y. M., Ye P. Z. & Wang S. Multiple magnetic clouds: Several examples during March - April, 2001. J. Geophys. Res. 108, 1370 (2003).

[b15] Kaiser M. L. *et al.* The stereo mission: An introduction. Space Sci. Rev. 136, 5–16 (2008).

[b16] Louge M. Y. & Adams M. E. Anomalous behavior of normal kinematic restitution in the oblique impacts of a hard sphere on an elastoplastic plate. Phys. Rev. E 65, 021303 (2002). 10.1103/PhysRevE.65.021303 11863512

[b17] Kuninaka H. & Hayakawa H. Anomalous behavior of the coefficient of normal restitution in oblique impact. Phys. Rev. Lett. 93, 154301 (2004). 15524884 10.1103/PhysRevLett.93.154301

[b18] Kuninaka H. & Hayakawa H. Origin of rebounds with a restitution coefficient larger than unity in nanocluster collisions. Phys. Rev. E 86, 051302 (2012). 10.1103/PhysRevE.86.051302 23214774

[b19] Shen F., Shen C., Wang Y., Feng X. & Xiang C. Could the collision of cmes in the heliosphere be super-elastic? validation through three-dimensional simulations. Geophys. Res. Lett. 40, 1457–1461 (2013).

[b20] Temmer M., Veronig A. M., Peinhart V. & Vršnak B. Asymmetry in the CME-CME interaction process for the events from 2011 February 14-15. Astrophys. J. 785, 85(7pp) (2014).

[b21] Lugaz N., Farrugia C. J., Manchester W. B. IV & Schwadron N. The interaction of two coronal mass ejections: Influence of relative orientation. Astrophys. J. 778, 20(14pp) (2013).

[b22] Mishra W., Srivastava N. & Chakrabarty D. Evolution and consequences of interacting CMEs of 9-10 November 2012 using STEREO/SECCHI and *in situ* observations. Sol. Phys. 290, 527–552 (2015).

[b23] Chané E., Jacobs C., Van der Holst B., Poedts S. & Kimpe D. On the effect of the initial magnetic polarity and of the background wind on the evolution of CME shocks. Astron. & Astrophys. 432, 331–339 (2005).

[b24] Feng X., Wu S. T., Wei F. & Fan Q. A class of TVD type combined numerical scheme for MHD equations with a survey about numerical methods in solar wind simulations. Space Sci. Rev. 107, 43–53 (2003).

[b25] Feng X., Xiang C., Zhong D. & Fan Q. A comparative study on 3-d solar wind structure observed by Ulysses and MHD simulation. Chinese Sci. Bull. 50, 672–678 (2005).

[b26] Shen F., Feng X., Wu S. T. & Xiang C. Three-dimensional MHD simulation of CMEs in three-dimensional background solar wind with the self-consistent structure on the source surface as input: Numerical simulation of the January 1997 Sun-Earth connection event. J. Geophys. Res. 112, A06109 (2007).

[b27] Shen F., Feng X. & Song W. B. An asynchronous and parallel time-marching method: application to the three-dimensional MHD simulation of the solar wind. Science in china Series E: Technological Sciences 52, 2895–2902 (2009).

[b28] Wu S. T. & Wang J. F. Numerical tests of a modified full implicit eulerian scheme with projected normal characteristic boundary conditions for MHD flows. Comput. Methods Appl. Mech. Eng. 24, 267–282 (1987).

[b29] Hayashi K. Magnetohydrodynamic simulations of the solar corona and solar wind using a boundary treatment to limit solar wind mass flux. Astrophys. J. 161, 480–494 (2005).

[b30] Wu S. T., Wang A. H., Liu Y. & Hoeksema J. T. Data driven magnetohydrodynamic model for active region evolution. Astrophys. J. 652, 800–811 (2006).

[b31] Lepping R. P. *et al.* The Wind magnetic field investigation. Space Sci. Rev. 71, 207–229 (1995).

[b32] Ogilvie K. W. *et al.* SWE, a comprehensive plasma instrument for the Wind spacecraft. Space Sci. Rev. 71, 55–77 (1995).

[b33] Ciaravella A. *et al.* Physical parameters of the 2000 February 11 coronal mass ejection: Ultraviolet spectra versus white-light images. Astrophys. J. 597, 1118–1134 (2003).

[b34] Kumar A. & Rust D. M. Interplanetary magnetic clouds, helicity conservation, and current-core flux-ropes. J. Geophys. Res. 101, 15667–15684 (1996).

[b35] Wang Y., Zhou Z., Shen C., Liu R. & Wang S. Investigating plasma motion of magnetic clouds at 1 AU through a velocity-modified cylindrical force-free flux rope model. J. Geophys. Res. 120, 1543–1565 (2015).

